# Embodied, Exploratory Listening in the Concert Hall

**DOI:** 10.3390/bs15050710

**Published:** 2025-05-21

**Authors:** Remy Haswell-Martin, Finn Upham, Simon Høffding, Nanette Nielsen

**Affiliations:** 1 London College of Music, University of West London, London W5 5RF, UK; 2 RITMO Centre for Interdisciplinary Studies in Rhythm, Time and Motion, University of Oslo, 0318 Oslo, Norway; shoeffding@health.sdu.dk (S.H.); nanette.nielsen@imv.uio.no (N.N.); 3 Department of Sports Science and Biomechanics, University of Southern Denmark, 5230 Odense, Denmark

**Keywords:** concert research, audience member, exploratory behavior, respiration, embodied participation, enactivism, ecological psychology, expertise, aesthetic experience, affect, attention

## Abstract

Live music can afford novel, transformative aesthetic interactions for individual audience members. Nevertheless, concert research tends to focus on shared experience. In this paper we offer an account of exploratory listening that foregrounds embodied–enactive engagement and affective resonance through close analysis of the music, physiological measurements, and reflections from interviews. Our analysis centres on data collected from two musician audience members about one specific piece out of a larger interdisciplinary project involving concerts given by the Stavanger Symphony Orchestra and The Norwegian Radio Orchestra in March and June of 2024. Through the combination of in-depth phenomenological interviews with musically skilled audience members and measurements of breathing and body motion, we explore aesthetic enactment beyond common patterns of ‘synchronised’ response, focusing on audience members’ experiences of Harald Sæverud’s ‘Kjempeviseslåtten’ (The Ballad of Revolt) (1943). We find forms of absorbed, both imaginative and embodied involvement, of listeners enacting meaningful contact with, and pathways through, the music that in some ways corroborate crowd patterns but also reveal exploratory expertise and idiosyncratic affective orientations.

## 1. Introduction

### 1.1. Background

Live orchestral concerts can exert a strong pull and push on audience members’ attention, affective interpretation, and imaginative exploration. Live concerts make demands for personalised connection to music while being framed by social affordances, bodily co-presence, and expectations for particular listening behaviours (Pitts, 2005; Wald-Fuhrmann et al., 2021). The concert hall is a site of musical absorption (Swarbrick et al., 2024), where listeners can experience powerful forms of altered consciousness and senses of self, space, and time (Høffding et al., 2025). It is equally a space where the mind can wander in ways, sometimes coupled with, yet at other times, unrelated to the music. And all of this unfolds in—*through*—time. In his work on contemporary performance, Martin Barker points out that (a)live experiences are emergent experiences: ‘that is, as they are experienced, they are felt to grow, to integrate, and to open up new possibilities. This is the sense and way in which they are transformative’ (Barker, 2016, p. 29). Liveness always carries the potential for novelty, even in performances of familiar music, and even for expert listeners. All of this suggests that audience members’ experiences should be unique and distinct for personal and performance-specific reasons.

In this article we focus on a particular site of exploratory, aesthetic interaction: the classical concert hall. The work presented here comes out of the Bodies in Concert^1^ project based at the RITMO Centre for Interdisciplinary Studies in Rhythm, Time, and Motion (University of Oslo). Contributing to recent interdisciplinary directions in concert research (Dobson & Pitts, 2011; Dearn & Pitts, 2017; Merrill et al., 2023; Pitts & Price, 2021; Tröndle & Bishop, 2021; O’Neill & Egermann, 2022; Swarbrick & Vuoskoski, 2023), the larger project explores musician and audience behaviour and experience using a range of qualitative and quantitative methods: physiological measurements and motion capture from musicians and audience members across repeated performances, audio visual recordings from many angles, and phenomenological interviews with select participants. In this paper, we combine qualitative phenomenological inquiry and the analysis of physiological data to investigate audience member engagement with a specific musical work performed at concerts given by the Stavanger Symphony Orchestra (SSO) and the Norwegian Radio Orchestra (KORK). This combination lends the study a novel, ‘data rich’ (Reybrouck et al., 2024) vantage point from which to observe embodied forms of spectatorial intentionality and perception–action sequences that are typically hidden from view in a setting known for stillness and silence. Rather than looking for what is common across an audience, we instead connect individual accounts of listening experience with bodily measurements taken during the live performance.

Interdisciplinary collaboration of this kind also helps us account for the experiential richness of concert spectatorship, including its variability and uncertainty. The risk of attending live performances was highlighted by one of the skilled musician audience members we interviewed:

Something I really like about live music is that it involves some sort of risk. It may not be perfect and I really like that possibility…someone could play the wrong note, or something could happen in the room, a distraction or, I don’t know, a microphone could tip over or something. Yeah, I’m not sure, but I think you have to be there, right? You have to be right there, right then to experience that moment. And that’s my favourite thing about live music.

Being there as an audience member is to be involved, to share in this risk in some way. The experiential accounts and bodily behaviours of the musically trained audience members analysed in this study reveal involved, invested listening. While our skilled audience members are unable to participate in their musician roles, and conform to classical concert expectations for restrained spectatorship, we see active, exploratory interaction with bodily performances on stage that they—as listeners and performers—themselves know from creative practice. Exploratory contact with the music, we argue, is a negotiation between this embodied musical knowledge and their role as audience members.

In what follows, we first situate our study within an ecological–enactive theoretical framework. The dynamic encounter with music—*musicking* (Small, 1998)—in the concert hall is complex; our theoretical approach in this article is designed to meet the challenge of accounting for its ontological and epistemological richness. We draw on recent concert research which has argued that an ecological–enactivist approach to the concert experience convincingly embraces the dynamic complexity of that experience while providing a framework with robust explanatory power (Martin & Nielsen, 2024). The ecological–enactive view presented here complements recent philosophical work which emphasises and explains the embodied nature of attention, nesting perceptual agency with enactivist theories, arguing that embodied mental action plays a role in structuring the field of experience (Bruineberg & Stone, 2024). Theoretical framing is followed by the Methods and Materials section, where the experiments are outlined and the case study selection is explained. In Section 3, an analysis of the case studies is presented. In the Discussion section, we connect case study results with the larger context and theory.

### 1.2. Theory

Our study of exploratory listening in the concert hall is guided by several complementary theoretical threads from ecological psychology, enactivism, and phenomenology (Clarke, 2005; Matyja & Schiavio, 2013; Read & Szokolszky, 2020; Gallagher, 2011, 2017), which inform perspectives on musical exploration. More generally, an ecological orientation foregrounds perceptual exploration and action in specific (social, cultural, material) environments. Eric Clarke, in his application of James J. Gibson’s ecological theory to the perception of music, has compared the perceptual view with that of ‘information-processing’. Clarke notes that information-processing appears to be a disembodied process where perception ‘is treated as a kind of disinterested contemplation, with no connection to action.’ (Clarke, 2005, p. 15) The following is offered as a summary of the ecological alternative: ‘When humans and other animals perceive the world they do so actively. Perception is essentially exploratory, seeking out sources of stimulation in order to discover more about the environment…Actions lead to, enhance, and direct perception’ (Clarke, 2005, p. 19). In the context of music, then, the ecological perspective encourages the researcher to think about what musical structures, settings, and practices afford, and what opportunities they offer for active, meaningful engagement and interaction.

In the ecological view, the ongoing exploratory striving for resonant contact with environments shapes action–perception sequences. But how is it that we come to know environments, including musical environments, in such a way that we can orient ourselves around them meaningfully? Ecological theory points to the refinement of perceptual skill and intelligence over time. ‘Perceptual learning’, developed in the work of Eleanor J. Gibson, is the key concept here (Gibson, 2000). Applying this idea directly in the context of music, Simon Zagorski-Thomas conceives of perceptual learning as involving the creation of ‘well-trodden pathways’ (Zagorski-Thomas, 2014, p. 8) in thinking and action. There is a practical utility to such development, but perceptual learning involves changes in action–perception sequences that enable the discovery of meaning (Gibson, 2000). Our study addresses how musically trained audience members track and traverse pathways through live music as it unfolds. There is ample evidence of embodiment informing music perception in trained musicians (Zatorre et al., 2007), and here we extend this relationship physiological with measurements and experiential reports.

In his recent article ‘What does Musicking Afford?’ (Clarke, 2024), Clarke considers ecology in relation to philosophical aesthetics. After noting the continued popularity of (Kantian) disinterested contemplation in aesthetic theory, Clarke instead makes the case for an ecological aesthetics of attention that acknowledges our ‘*interested* or invested perspective on the world’ (Clarke, 2024, p. 56). The interdependence of perception and action is once more foregrounded. It is to actual concrete actions that Clarke turns in the paper’s main case study, which explores viola performance in an informal concert setting. Initially, however, Clarke briefly considers the kinds of action afforded in concert-hall and headphone listening, ‘which seems to be passive’ (Clarke, 2024, p. 58). It is noted, however, that ‘virtual’ action—i.e., the mental tracing of musical (including tonal) movements and patterns—is a possibility. This calls to mind the ‘paradox of passivity’, as Reason et al. put it, in aesthetic reception, where ‘sometimes the most active role is to do nothing physically and yet be central to everything’ (Reason et al., 2022, p. 7). But more overt enactment of musical movement is also possible, for example where a skilled listener, on detecting a tension or suspension in the music, ‘leaps up to the piano and resolves it’ (Clarke, 2024, p. 58). These brief examples point, then, to listening not as passive or detached but as about potentially rich, dynamic interactions within—to recall Alan Costall’s summary of the ecological view—an ecology of embodied agency (Costall, 2007). Ecological agency marks a serious challenge to stimulus–response frameworks, simplistic accounts of causality, and the sense that we live our lives ‘*just waiting for things to happen to us*’ (Costall, 2007, p. 67, emphasis in original). Costall charts Gibson’s relational reorientation, with a nod to Merleau-Ponty’s phenomenology, to note that ‘once we allow for the active nature of human beings and other animals, the “stimulus” can no longer figure as an efficient cause, nor be considered as the starting point for perceiving’ (Costall, 2007, p. 66). Ecological exploration involves skilled, intelligent, embodied perception including the effective ‘foreseeing’ (Costall, 2007, p. 73) of affordances. This foundational claim points towards the role of anticipation and preparation in our bodily relationship with, and being-in, the world (see also, for example, Gallagher’s discussion of anticipatory kinaesthesia in (Gallagher, 2011)). In short, perceptual readiness and agency is not the work of a purely mental prediction machine, but involves an engaged body (Bruineberg & Stone, 2024).

Through an enactivist lens, Høffding and Schiavio summarise this in terms of a ‘dynamic unity’, where ‘perceiving and doing become two complementary aspects of the same experiential dimension’ (Høffding & Schiavio, 2021, p. 812). Here too, perception is understood as involving a knowledgeable ‘bodily grasping’ (Høffding & Schiavio, 2021, p. 812) on the part of the embedded agent (Noë, 2006; Di Paolo et al., 2017; Fuchs, 2017). It is within this framework that they conceive of (mental and physical) exploratory behaviours in music creation. Skilled adaptation in specific performance contexts is a central theme, as is participatory sense-making that arises not purely from internal mental computation, but from sensorimotor exploration that integrates a range of ‘extra-neural’ (cultural, material, technological) resources (Høffding & Schiavio, 2021, p. 814). The saxophonist, Torben Snekkstad’s free improvisation is offered as an example of exploratory music-making. The authors observe that a dual intentionality arises from (1) technical exploration of the musical possibilities which, in an ensemble context, are necessarily relational; and (2) *exploratory expertise*—that is, expertise from accumulated practice as enabling the exploration of music through multiple integrated perspectives—enacted through mental strategies that alter time-consciousness and, with this, the musical performance. Our study reorients this work, interpreting the exploratory expertise of musicians who are not performing, but are participating as audience members. Such expertise underpins forms of intentionality involving technical appraisal and tracking of the music, as well as reflections on self-involvement and embodied interaction. The claim here is that their practice enhances sensitivity to musical phenomena and allows flexible engagement as both player and spectator.

Performing arts audience research substantiates this. In a highly relevant publication, Matthew Reason and Dee Reynolds analyse audience interviews and group discussions and highlight the role of ‘embodied anticipation’ (Reason & Reynolds, 2010) in dance spectatorship. This is evidenced in reports of physiological transformation—including of breathing—and senses of muscular tension as audience members track unfolding choreography. This informs an active, affectively rich, pleasurable, mode of engagement Reason and Reynolds conceptualise as ‘kinesthetic empathy’. Their account of watching dance also points to a multifaceted intentionality. Experiences of being ‘drawn in’ by dance performance also involved imaginative participation (of ‘doing it’, of being ‘with it’) and, in some reports, critical self-reflection (‘I couldn’t do that’). This exploratory ‘bridging of the gap’ (Reason & Reynolds, 2010, p. 60) between spectator and performer is evidenced in the skilled concert hall listening case studies presented below. Brain imaging research substantiates this connection between perceiving and doing specifically through music (Zatorre et al., 2007). Expert musicians and newly trained musicians show premotor activity in reaction to auditory stimuli related to their practiced musical actions.

## 2. Materials and Methods

We employ an exploratory, mixed method approach that combines interview data and measurements of breathing and body motion, focusing on two audience members out of the larger dataset from the Bodies in Concert experiments.

### 2.1. Bodies in Concert Experiments

The Bodies in Concert project is a series of collaborations between RITMO and professional orchestras to measure the bodily involvement of musicians and audiences during live performance. The two experiments drawn from here are each from performances in 2024.

With the Stavanger Symphony Orchestra (SSO), we measured aspects of the performance (audio and video recordings), some of the audience, and members of the orchestra during the dress rehearsal and during seven performances of a children’s concert program (LYDO). On our request, ‘Kjempeviseslåtten’ was included in the concert program, presented in shortened form by starting from m. 50 in the orchestral arrangement of this famous Norwegian work. Thirty-one members of the orchestra volunteered to wear Equivital sensor vests under concert clothing, some also participating in rating tasks after two performances and additional interviews about their experience of a specific work. Twenty music students were recruited from a nearby university to attend as audience members at one of two of these concerts while wearing the same sensor vest, completing questionnaires after each musical piece. The first concert these music students attended was a performance to an audience of school children (with approximately 500 children in the auditorium) and the second was a ‘family’ concert of similar audience size, with a mixture of adults and children. Eleven of the music student audience members additionally participated in phenomenological interviews later that day or the next day.

With the National Radio Orchestra of Norway (KORK), we similarly measured aspects of the performance, audience members, and orchestra members during rehearsals and the performance of a special collaborative concert with a science radio program. Related to our interest in performer coordination, the orchestra included in the program two performances of ‘Kjempeviseslåtten’ as arranged for orchestra in full, opening the concert with the concert master conducting on the podium, and ending the show with the same work performed without a conductor, as they normally perform it. During this concert, 54 members of the orchestra work wore Equivital sensor vests, including the conductor/concert master, and some additionally participated in interviews between rehearsals. Five music students were recruited from local music schools (university level) to attend the performance while wearing the same sensor vests, completing similar questionnaires to the SSO music student audience members, and participated in the phenomenological interviews directly after the concert or the following morning. The ticketed audience were also offered a chance to wear similar sensors through the performance (Equivital vests or Movesense cardiac belts with additional accelerometers), to answer a different short set of questions in the concert pamphlet, and to wear reflective wristbands for hand motion tracking with an additional infrared camera system recording the audience.

### 2.2. Interviews

The ‘phenomenological interviews’ for this study were between 35 and 65 min in length and were conducted by three researchers. The interviews were recorded using an institutional server (Nettskjema Diktafon 5.0.8) and initial transcriptions were generated using Autotekst software (https://autotekst.uio.no/en). Transcripts were reviewed for accuracy by a research assistant.

Interview data collection and analysis employed a conceptual framework for understanding audience members’ experiences, integrating semi-structured interviews and phenomenological theory (Høffding & Martiny, 2016). This can be situated in the context of a collection of related methods that seek to empirically use the second-person experience (as distinguished from first-person methods relying solely on abstract thinking or formal logic, and third-person methods relying on measurements and experiments). A useful overview of some key methods—including ‘Interpretative Phenomenological Analysis (IPA), ‘Micro-Phenomenology’, ‘Cognitive Ethnography’, and ‘Phenomenologically Grounded Qualitative Research (PGQR)’—is provided in Høffding et al.’s recent Phenomenology and Cognitive Sciences special collection editorial (Høffding et al., 2023). These methods, it is noted, vary in their allegiance to classical phenomenology, their application of key concepts, and their approach to the collection and analysis of qualitative data. With this, they vary in their suitability to address target experiences with varying qualities and durations, ranging from seconds to years. Our interview protocol and analysis are guided by Phenomenologically Grounded Qualitative Research (Køster & Fernandez, 2023), where phenomenological concepts are frontloaded in the design of semi-structured interview guides and are applied in the interpretation of interview data.

Interviews were analysed through a two-phase phenomenological protocol (Høffding et al., 2025). The first, “emic” phase involves the close reading of interview transcripts and the identification of salient themes. In phase two, the more deductive “etic” phase, phenomenological concepts and theory are re-integrated to support an analysis of salient codes. Cross-coding was conducted by researchers to ensure consistency. Agreed emic themes include ‘modes of listening’, ‘mental imagery’, ‘(shared) embodiment’, ‘interaction with the music’, ‘attention’, ‘absorption’, and ‘strong emotional experience’, all of which speak to exploratory listening in the concert hall. Within ‘modes of listening’, sub-themes of ‘analytical listening’, ‘professional listening’, and ‘appraisal of musical expression’ are very relevant to listeners’ skilled interactions with the performance and facility for exploring the music as it unfolds.

### 2.3. Case Study Selection

The process of selecting case studies out of this extensive dataset should be understood in the wider context of Bodies in Concert. Based on previous work, ‘Kjempeviseslåtten’ was chosen to be included in both concert programmes and for interviews of audience members to enable lines of inquiry on the piece specifically. During the coding process, instances of detailed experiential reports on the piece were then identified. In these reports, we found two participants with experience performing this work in different contexts and, as such, their reports, and appraisals of the music, were framed and guided by an expert understanding of the technicalities of performance. This understanding also affords specific embodied relations to the measured musicians performing on stage. From there we proceeded to a detailed comparison between interview accounts and physiological measurements. The two case studies explored here involve participants that were, at the time, studying music at a university or music conservatory in Norway, and thus are skilled in terms of musical knowledge and performance capability. Their stylistic and disciplinary focus (i.e., performance, conducting, composition) is distinct, however.

### 2.4. Physiological Measurements

The Equivital sensor vests are designed and programmed to collect multiple physiological signals simultaneously, each logged at their own rate with internally generated timestamps: respiration wave (26.5 Hz), core body motion in three-dimensional accelerometry (256 Hz), 2-line electrocardiogram (256 Hz), and skin temperature (0.07 Hz).

The respiration wave captured by this sensor system is a relative measurement of stretch in a single belt around the ribcage, positioned a bit below the pectoral muscles. Designed to capture the oscillation of inspiration and expiration across wide range of postures and activities, the reported signal values are produced with an adapted sensitivity, retaining not absolute stretch but rather variation around a stable mean with range adjustments over minutes (slowly increased sensitivity) or in reaction to signal clipping with extremely large breaths (rapid decreased sensitivity). The resultant wave is not reliable for calibrated estimates of lung volume or air flow; however, it is usable for assessing phase onset timing (inspiration and expiration) and some qualities of breath shape that distinguish categories of respiratory activity and control mechanisms.

In the following analysis, audience participant respiration is classified per breath as quiet or disrupted. Behaving typically for audience members of classical music concerts, these participants were not vocalising or performing, demanding physical exertion, while music was being performed. Under such circumstances, it is expected that healthy human adults default to quiet breathing: a discrete, efficient, and relatively stable pattern of respiration with an active shallow inspiration of around 1s duration immediately followed by elastic expiration to a stable lung volume and a short pause before the next inspiration (Upham, 2018). Quiet breathing tends to have respiratory periods of around 3 or 4 s. This kind of breathing is also easily disrupted by deeper and longer breaths, like spontaneous augmented breaths (3 or more times the depth of quiet breaths (Vlemincx et al., 2013)), or shorter and deeper inspirations in support of controlled expiratory actions, like vocalisations or timed limb movements, whether performed or sometimes imagined (Conrad & Schönle, 1979). The influence of imagined actions, such as inner spontaneous speech on concurrent respiration, is documented but not extensively studied. To facilitate the identification of respiratory disturbances in audience member measurements, each recorded respiratory cycle was evaluated for classification as quiet or disrupted. A breath was counted as quiet if it fell within specific thresholds set to capture sequences of this distinctive mode of respiration, using features of absolute phase durations, relative depth, and change in duration and depth from the previous cycle. These thresholds were established on respiration recordings from Bodies in Concert datasets without adjustment for specific participants or pieces. Treatments of these sequences were conducted using functions from the forthcoming respy Python library v1.0.0 and shared in the published code repository (Upham, 2025).

Quantity of motion time series for participants’ core body were extrapolated from the accelerometer measurements via instantaneous jerk magnitude after downsampling measurements to 25 Hz.

### 2.5. Music and Music Analysis

Harald Sæverud’s ‘Kjempeviseslåtten’: Canto Rivoltoso per orchestra, Opus 22a nr. 5. ‘Kjempeviseslåtten’ (The Ballad of Revolt) (1943) is symbolic of the resistance and dedicated to the ‘Home Front’s big and small fighters’; the piece is generally familiar to Norwegian audiences and carries rich potential for affective and imaginative involvement on the part of the listener. It begins with musical illustrations of the sounds of war, eerie suspenseful droning with interrupting fanfares, fugal confusion, and explosive hits. The second part of the piece presents repetitions of a folk melody, progressively rearranged to grow from solo strings over a quiet drone to a climactic bombastic tutti. The score studied was the Musikk-Huset A/S Noreg-Edition No. 32a, the same edition as used by the performing orchestras. Time stamps corresponding to each instrumentalist’s entries, rehearsal numbers, and structural sections were identified in audio recordings of each performance using Sonic Visualiser (Cannam et al., 2010). The performance by the SSO, in the first case study, was an excerpt of the larger piece, starting at rehearsal number 5 (m. 51), presenting only the repeated folk melody. To facilitate comparisons between the two versions performed by KORK in the second case study, dynamic time warping on the audio of each performance was used to align physiological signals and event time stamps from the second performance into the timing of the first (Prätzlich et al., 2016). Evaluation of the music and physiological measurements were initially performed in Sonic Visualiser before relevant annotations were extracted and plotted using Python notebooks (Upham, 2025). Audio of the performances are displayed throughout as constant Q transform spectrograms (McFee et al., 2023), with a hop size of roughly 23ms. This spectrogram presents energy per half step interval, corresponding to pitches from C1 to C7, a representation similar to a piano roll that facilitates the recognition of musical information.

## 3. Analysis

### 3.1. Case Study 1

**Figure 1:**
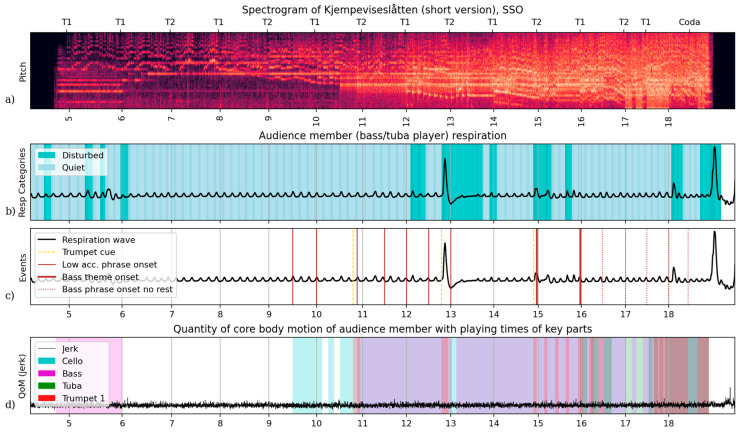
Figure of orchestra audio and annotated audience member respiration and quantity of motion for the full duration of the SSO’s performance of ‘Kjempeviseslåtten’ for Case Study 1. (**a**) A Constant Q-transform spectrogram of a professional stereo audio recording of the orchestra performance, showing energy per half-step band over seven octaves (C1–C8). Above are structure markers indicating the start of each theme’s statement (T1, T2), and below are rehearsal numbers according to the full score (1–4 were cut from this performance). (**b**) The full respiratory wave as captured by the Equivital sensor vest on this audience member (relative chest stretch). Each respiratory cycle is categorised as quiet or disturbed based on the duration of phases and deviation from the previous cycle. (**c**) The same respiration wave as above, with salient moments marked with vertical lines according to the legend. (**d**) The audience member’s concurrent quantity of motion (magnitude of jerk) with intervals of active play for salient parts according to the score: Cello, Bass, Tuba, Trumpet 1.

Our first case study explores the experience of a university-level music student who attended the SSO LYDO concert held on 8 March 2024. Figure 1 shares this participant’s respiratory sequence and quantity of motion during the piece as performed by the SSO along with related performance annotations. This audience member’s respiration was categorised as quiet breathing thought 78% of this piece, a little above the median (70%) for audience members during SSO’s performance of ‘Kjempeviseslåtten’. This participant’s quantity of motion was low and stable until they began to applaud after the final note.

This particular audience member is a tubist and double bass player who reported a very particular autobiographical attachment to ‘Kjempeviseslåtten’. The affective significance of ‘Kjempeviseslåtten’ is explicated in their admission that the piece ‘has always had a special place in my heart because I played in the military band and it felt very dramatic because it has something to do with the war…he wrote this piece to honour them, the fallen Norwegians’. In the interview, specific past performances were mentioned, including ‘feeling the connection between the soldiers of 1945 and meeting a soldier in 2015 when I played [‘Kjempeviseslåtten’] the first time’. Yet despite playing the piece ‘hundreds of times’ in a wind band setting, this was the listener’s first time hearing the piece performed live by a full orchestra, including the large string section, which gives the piece an altogether different affective profile. The listener’s reflection on this involved consideration of the timbral and dynamic properties of the performance in comparison to their experience of it in a wind band setting:

It is a very different and more emotional sound than you would get from a wind band…there’s something about the wind band which play this piece which can tend to be very harsh or very loud. And with the strings in an orchestra you get a way more warmer sound out of it and I think that is something that, you know… the sound in itself was making this piece even more, more ‘grave’, like very serious.

**Figure 2:**
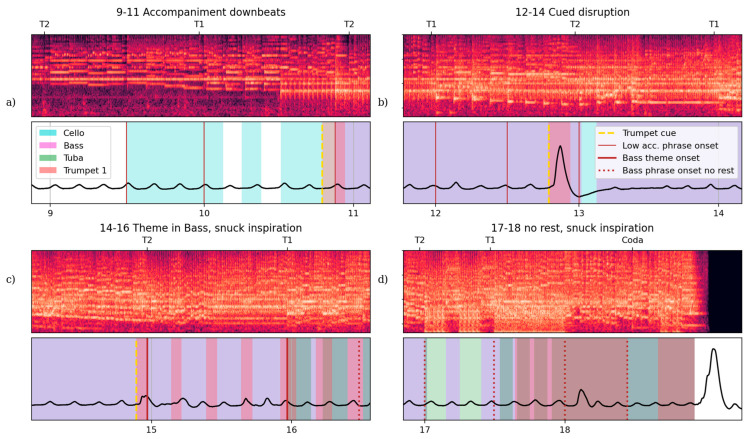
Four 40 s excerpts of performance audio and audience member respiration from Case Study 1, with instrument parts and event annotations from Figure 1c,d overlayed on the respiration wave. (**a**) Excerpt rehearsal numbers 9–11 (79–119 s) show the solo cello entry going into the low strings playing accompaniment and audience member inspirations preceding bassline phrase onsets. (**b**) Excerpt 12–14 (130–170 s) shows a large respiratory disruption (rapid augmented inspiration) directly following the trumpet fanfare cue for a section that introduces a more complex arrangement of melodic content partially shared with the basses (13). (**c**) Excerpt 14–16 (170–210 s) shows thematic statements when the basses are playing the melody, again with one phrase (15) cued by a trumpet fanfare. (**d**) Excerpt 17–18 (215–255 s) shows more instances of disrupted inspiration through the final theme’s bombastic theme statements, including bass and tuba passages performed *fff* that do not pause for breaths as often as a tuba player might need.

Of particular interest is the listener’s relationship to a specific section of the orchestra. Special attention to the double bass section was reported in the interview: ‘I guess you get kind of obsessed with the players, who will be playing what and so on when you know a piece…I’ve been playing it both for tuba and the double bass so I was watching the double basses a lot, especially into the ending of the piece’.^2^ Particular passages were identified in the interview, notably the descending bass sequence at the first theme (T1) after rehearsal number 17 (Figure 1a and Figure 2d). Close analysis not only reveals an attentional connection to (and an ability to recall) these sections of the orchestra; a bodily relationship is also evident, notably in the listener’s breathing profile, which indicates an idiosyncratic embodied orientation of ‘Kjempeviseslåtten’ characterised by anticipation and physical preparation. This audience member is not breathing like a performing musician (Figure 1b), for most of the piece they are breathing quietly, a little more stable (78%) than the median ratio (70%) for audience members to the SSO performances of this work. Nor did they move like a performing musician: their core body motion showed no shifts of posture to match entries of key parts or jolts of limb motion until the applause (Figure 1d). However, the timing of their breathing and some instances of disruption from quiet breathing align at moments salient to the parts they reported practicing previously and attending to during this performance.

For instance, there are well-timed inspirations (active phase of quiet breathing) before many low strings entries and phrase beginnings (Figure 1c and Figure 2a,b). In the excerpt of Figure 2a without any active expiration phase, this audience member’s inspirations were timed to allow expirations with the beginning of these lines that start on downbeats against the melody pickup (T1, T2) between rehearsal number 9 and 11. The trumpet fanfare before 11 cues the bass’s entry before T2. Excerpt 12–14 (130–170 s, Figure 2b) shows continued expiration alignment to accompaniment onsets and a large respiratory disruption (augmented breath) directly following the trumpet fanfare cue for a section that introduces more complex arrangement of melodic content partially shared with the basses (13). In Figure 2c, this audience member’s expirations shift and align with the bass part pickup rather than the downbeats. An audience member aligning expiration onsets with this handful of bass part entries is a possible coincidence, but this is unlikely, and not a shared with any other audience members at this performance (see Supplementary Materials).

In the above cases, the bass part would grant a tuba player time to inhale before playing a line; however, in Figure 2d, these parts play through phrases. Here, this audience member’s quiet breathing expirations are not aligned with the phrase structure. Their account of attention to this line, and their reported experience with performance of this music, likely facilitated this distinct embodied engagement with the music, as they unconsciously prepared for anticipated entries when possible, even if the inspirations were too shallow for actual playing.

The listener also demonstrates aberrantly quick inspirations in two places, when a low brass would sneak a breath from an unbroken line. In Figure 2c, the bass phrase onset before 15 is prepared by a disrupted inspiration that is unusually fast, timed to sneak a breath between notes when the line offers no rests for a tubist to inhale. Through the end of the piece, Figure 2d, these low parts (Bass and Tuba) do not allow for breaths that a tuba player might need for these long phrases to be performed fortissimo or louder. After 18, a disrupted inspiration by this audience member is quick and sharp like before 15 (Figure 2c), here timed with the dot of the main melody played in unison across the whole orchestra. This moment is perfect cover for a snuck breath before the final fanfare.

A sensitivity to performance physicality is enacted in snuck breaths, which suggest a readiness for the challenge of making it through long loud passages. These two instances come from intervals of continuous play lasting 25 and 13 measures, respectively. This behaviour is consistent with this participant’s reported attunement to the physical demands of performing this music: ‘my teacher told me when we did this downward scale thing that I had to imagine I was a gorilla playing, to get the right posture…it impresses me when you see musicians play something I know is really difficult.’ Thus, the audience member seems corporeally invested in the performance, in a very particular way, as it unfolds. Like adjustments to quiet breathing timing, action–perception sequences are guided by a focussed transposition of skilled, performative knowledge. This audience member also takes a very large, augmented breath once the piece ends, as performing musicians often do, a gesture that commonly marks a release of conscious respiratory control.

In summary, physiological measures show correspondence to the audience member’s recounted expert exploration of this performance, combining expert, embodied knowledge and perceptual sensitivity from tuba practice, observed bass playing, and autobiographical associations.

### 3.2. Case Study 2

Our second case study explores the experiences of an audience member who attended the KORK concert in Oslo on 6 June 2024, at which the full version of ‘Kjempeviseslåtten’ was performed twice, at the beginning with the concert master conducting and at the end of the program without a conductor (as this orchestra often performs it).

**Figure 3:**
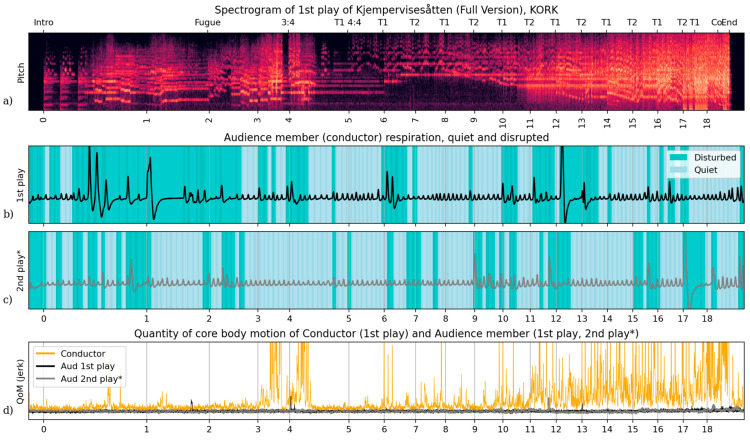
The full Case Study 2 measurements and performance audio from KORK’s first performance of ‘Kjempeviseslåtten’ with annotations, *signals warped to first performance time. (**a**) Constant Q-transform spectrogram of a professional stereo audio recording of the orchestra, as in Figure 1. Above are structure markers including meter changes and theme onsets, and below are rehearsal numbers according to the full score. (**b**) The full respiratory wave of this case study audience member during the first performance (with conductor), categorised as in Figure 1b. (**c**) The full respiratory wave by this same audience member during the second play of ‘Kjempeviseslåtten’, with breaths categorised as in Figure 1b and plotted with time warped to align with the first performance. (**d**) Three quantity of motion measurements (core body jerk magnitude): the performing conductor during the first play, this audience member during the first play, and this audience member during the second play with time values warped like in (**c**).

At the time of the concert, they were studying conducting at the Norwegian Academy of Music, a university-level conservatory based in Oslo. We supplement the comparison of this participant’s accounts, motion, and respiration through two performances of the same work, with the motion and respiration from the conductor on-stage for the first performance. Figure 3 shows this audience member’s respiratory wave and motion in response to each performance in comparison to those of the conductor. Unlike in Case Study 1, this audience member shows less quiet breathing than most audience participants, 47% of the time compared to a median of 63% the of time through the first performance of this work, and 63% of the time compared to a median of 74% of the time through the second. However, in terms of quantity of motion, this participant was more on the low end relative to other measured participants, with relatively few spikes during the performances (Supplementary Materials). And as is evident in Figure 3d, the conductor’s core body motion far exceeds that of this, or indeed any, of the seated audience members.

As in the above case study, creative practice is a point of connection between this audience member and the concert repertoire, and they reported having previously conducted about half of the programme. They framed their attachment to ‘Kjempeviseslåtten’, more specifically, in terms of a ‘personal relationship’ forged through a particular experience of working on the piece: ‘I especially remember this masterclass I was at two years ago. And I was not doing a very good job. Or I don’t know if I was doing a good or bad job, but the teacher wasn’t very encouraging, he was very quick to comment negatively if you did something’. Elsewhere, it was suggested that the experience of ‘not doing a good job while conducting it’ at the masterclass may have shaped the affective profile of concert experience, noting ‘maybe I wasn’t so emotionally attached because of my background with this piece’. Through autobiographical association, this audience member shows a strong sensitivity to a specific performing perspective (i.e., that of the on-stage conductor) and a technical interest in the larger ensemble through the second performance. By their own account:

But I really liked when the second time they played it… I was thinking a lot on how hard it is to play this piece without the conductor, because there’s a lot of timing things that needs to be … they need to have a focal point… But I thought […] the focus of the musicians [was] much more to each other. And so they played those small melodies that they played within the group. They had much more freedom and they used the freedom to fill it with what they wanted to do. Which made me end up liking the second time better, even though the first one was cleaner.

With regard to the first performance, we were able to compare this audience member’s breathing and movement with that of the conductor. Conductor respiration is often adapted for communication with the ensemble (Fadiga et al., 2021) as well as supporting their physical exertion and affective experience. Our analysis exposes three patterns relating this audience member’s respiration and motion to that of the on-stage conductor.

**Figure 4:**
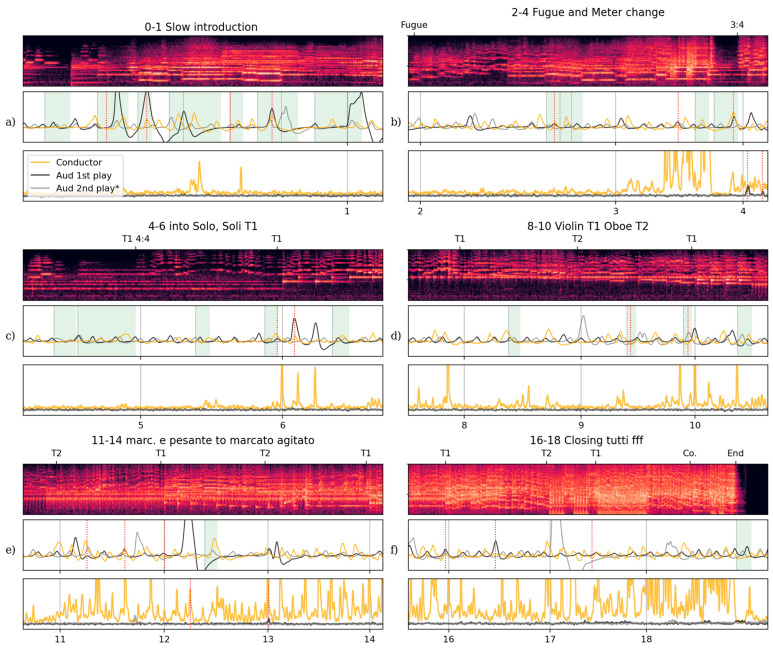
Six 60 s excerpts of performance audio, audience member and on-stage conductor respiration waves, and audience member and conductor quantity of motion, with additional annotations. (**a**) Excerpt 0–1 (15–75 s) from the slow introduction with green highlighting behind the respiration waves indicates held passages: intervals without new onsets and dotted red vertical lines indicate salient coincidences between the physiological measurements of the audience member and the on-stage conductor. (**b**) Excerpt 2–4 (109–169 s) shows the short fugue section and shift to 3/4 m with more held passages in green. (**c**) Excerpt 4–6 (185–245 s) shows the stillness going into the theme and variations, first played by solo viola and violin, then by full sections from 6. (**d**) Excerpt 8–10 (260–320 s) themes in variation, with the oboist playing the theme in 9–10. (**e**) Excerpt 11–14 (320–380 s) shows theme statements that vary in texture and expression with the Marcato agitato starting at 13. (**f**) Excerpt 16–18 (405–465 s) shows the closing section of this piece with near-tutti theme statements.

The first pattern concerns late deep breaths through held passages, intervals of a few seconds without new onsets, a behaviour evident in the on-stage conductor’s and in this audience member’s respiration sequence during both performances (Figure 4a–c). We see large deviations in breathing depth and inspiration timing, particularly through the first performance, which also often coincide with breathing phases of the KORK conductor. These are moments when, in order to stay in contact with the music, conductors seem to use breath to continue to feel and hold the passage of time, in the absence of clear temporal markers. A few instances of this can be observed in the spectator’s breathing during the opening of ‘Kjempeviseslåtten’. Before rehearsal number 1 (Figure 4a), this audience member showed substantial deep disrupted respiration during both plays through these held passages (highlighted in green), with coincidental inspirations or expiration onsets (dotted red lines) between the audience member and conductor towards the end of these passages. In a second excerpt starting directly before rehearsal number 4 (Figure 4b) the conductor and audience member breathing again shared expiration onsets towards the end of held passages, and also at a salient offbeat sting (after 3). The quiet passage before 5 (Figure 4c) has the audience member showing similar long disrupted breaths unaligned with the conductor. It is noteworthy that disrupted breaths occurred more frequently in the first (conducted) performance during held passages and, overall, certainly more frequently than observed in other audience members. This embodied feeling of time through deep breaths is a distinct means by which the spectator can explore musical time and space and interact with the performance.

The second pattern is that the timing of the audience member’s inspirations and expirations enact communicative functions of a conductor at important moments and indeed show a parallel to the on-stage conductor’s breathing. One example of this is a disturbed breathing timed with the conductor’s through the viola section playing of the main theme (Figure 4c). Audience respiration in the first performance shows disruption when the conductor takes control again for the soli theme statements (6), aligning in expiration onsets around their entry. This moment stands out as it is the first statement of the repeating theme played by a section rather than a soloist, and thus, when the conductor assumes expressive control of the ensemble. A second example is the sequence of inspirations that match those of the conductor before key wind phrase onsets (see Figure 4d). The audience member shows stronger respiratory alignment here during the second play with the conductor, specifically at crucial inspirations for the oboist playing the theme (9–10). These measurements also show a changing orientation in respiratory timing by conductors (on-stage and in the audience) to the breathing of the featured musicians over successive theme statements. The on-stage conductor’s and audience member’s expirations also coincide with the accompaniment’s accented downbeats through rehearsal number 11–12 (Figure 4e). This phase alignment between audience member (both performances) and conductor shifts the expiration against the metre to fall on the theme’s pickup in the variations through the closing passage (Figure 4f).

One further observation can be made about this listener’s core body movements’ relationship with the music. We find *distinct jolts of movement at salient, functionally demanding moments for a conductor*. These also serve to distinguish their first listening (with the on-stage conductor) from the second. One example of this relates to an off-beat cue after a change in metre (Figure 4b). In the body quantity of motion measurements, the audience member shows two jolts in time with the offbeat attacks (viola/harp/clarinet) after rehearsal number 4. A second example occurs at rehearsal number 13 (Figure 4e), as the theme statements change in texture and expression, moving suddenly with the change in expression at 13 during the first performance. This is the onset of marcato agitato, a cue of textural change—and thus expression from a more legato presentation—in the variation sequence. The audience member also shows a strong jolt of motion during the second playing at another accented downbeat (after 12, similar to expirations after 11). In plotting the on-stage conductor and audience member signals together, Figure 3 and Figure 4 shows how a seated audience member cannot breathe or move like a conductor for any extended period of time. However, there are many moments of alignment for this specific audience member, in respiratory phase, shape, and discrete jolts of movement. Some that align during both plays of this work and some that differ. These excerpts and annotations highlight alignments during salient conducting moments for this complex orchestral work: slow quiet passages, changes in metre and expression, shifts in focus between solos and sections, winds and strings, themes and accompaniment. We cannot claim that every instance of alignment is real expression of internalised conducting on the part of the audience member. The contrast in reported experience and measurements between the two performances demonstrate the audience member’s freedom from rehearsing the work of conducting this piece. Recall this audience member’s expressed contrast in focus and enjoyment, ‘liking’ the second performance more.

## 4. Discussion

The above cases illustrate idiosyncratic perceptual contact with, and interpretation of, the music that results from specific musical expertise. This is a richness overlooked by the common focus on average patterns across the audience in quantitative concert research. Audience members are enacting skilled knowledge of musical performance within the confines of acceptable classical audience member behaviour. Their listening, as captured through experiential reports and physiological measurements, does not reveal rote re-production of their playing experiences, but rather a negotiation of spectatorial and performative roles during each specific interpretation of ‘Kjempeviseslåtten’. Key to their exploratory expertise (Høffding & Schiavio, 2021) is their ability for skilled foreseeing (Costall, 2007) of salient performance actions, which is revealed through discrete behaviours. For example, adapting expiration timings during quiet breathing, which requires preparation with well-timed inspirations (the only active phase of quiet breathing) or jolts of active expiration and motion with accented beats. Some of these prepared alignments, though discreet, nonetheless suggest *expressive anticipation*.

Returning to our listeners’ experiential statements reveals the affective resonances of these embodied, exploratory interactions with ‘Kjempeviseslåtten’. For the listener in Case Study 1, the orchestral arrangement of the piece afforded a distinct interpretation of emotion, noting ‘the sound was making the piece even deeper for me’. Their expert sonic appraisal of the music offers one explanation for a heightened emotional power ascribed to the music. Another consideration is the nature of the perceptual contact with the music in this particular setting. While their ‘bodily grasping’ (Høffding & Schiavio, 2021) rests on active, anticipatory engagement, that they are an audience member in this setting perhaps alters their responsibility and attachment to it.

The tubist audience member’s interview also revealed intensive, selective attention to the relationship between the orchestra and the on-stage SSO conductor (a performer this audience member had worked with) at the end of ‘Kjempeviseslåtten’. Their familiarity with the ending of the piece, their technical understanding of how it is navigated in performance, as well as their admiration of the conductor shaped an affective transformation through the closing passages. The listener described the following:

At one point at the end I was getting a little stressed, because I know the piece is accelerating into the final bar and nothing was happening. And I saw she (the conductor) was also doing something really calmly with her arms and so I was watching her a lot, because I just love her technique…when I saw that she was trusting the [ensemble] I was getting really happy, really relaxed in the end. Because at some point she was just letting go of the band. So they did the acceleration by themselves.

This emphasises the strength of this audience member’s understanding of this piece of music, an understanding that facilitates multifaceted, multivalent experience. The moment described above is coincidental with an embodied attachment to—perhaps concern *for*—the performance which is evidenced by the snuck breaths (Figure 2c,d) and then relaxing into quiet breathing until the final note. The listener’s tracing and anticipation of specific (bass) musical lines, and awareness of the effort of performance, is driven by a skilled intentionality. At the same, the listener is freed from other demands of a playing situation, which opens exploratory opportunities for autobiographical recollection and novel affective trajectories. This form of expert exploration can be understood through the concept of ‘mind surfing’ (Høffding et al., 2024), which highlights the capacity for selective, intensive attention while simultaneously looping with imaginative and affective orientations.

Further consideration of our second case study, the conducting student, seems to support this line of thinking through the contrast in their experience of the two performances of ‘Kjempeviseslåtten’. This participant, generally, reflected on their habit of active, practical engagement when listening to music.

To me, music is a very, it can be a very active experience, so active listening I prefer, but it takes a lot of energy, because… as a musician…the more music I play the less I listen to music. I think not necessarily maybe because I find it boring or because it’s a job I think maybe for me music is as now synonymous with doing.

Their first listening featured an exceptional quantity of disrupted breathing activity (53%), often shaped and timed sympathetically with that of the on-stage KORK conductor—an expert enactment of ‘kinaesthetic empathy’, which bridges the gap (Reason & Reynolds, 2010) between their role as an audience member at this concert and their ‘outside’ experience as a conductor. Their matching deep and long breaths with the on-stage conductor during held passages suggests a shared enactment of musical time (Kozak, 2019).

The audience member’s embodied interaction with KORK’s second performance of ‘Kjempeviseslåtten’, performed without an on-stage conductor, retains a skilled sensitivity to the musical performance while showing less disrupted breathing and jolts of movement. This can be interpreted as a shift away from a practical, constrained experience, which, as mentioned above, is informed by self-critical recollections. The audience member described their second listening as ‘more analytical’, with a heightened awareness to musical detail which is not as common for them during ‘big’ emotional experiences. The capacity for active, analytical listening and emotional experience was framed by them in terms of the limits of ‘inward’ and ‘outward’ emotion and energy. The audience member described a necessary need to ‘distance myself somehow, or put my emotions outwards’:

When I conduct, I don’t have the surplus of energy to think inwards and outwards at the same time. But maybe that’s specific to me as a person. But I definitely will have an emotional reaction to a piece, but I don’t have the luxury of basking in the emotion because I’m doing something practical. And also because my job as a conductor is to stay connected to the musicians. If I’m connected inwards and not connected outwards, then I lose this connection, the connection between me and ‘the machine’ that I’m operating.

This statement affirms the claim that musicians’ internal experiences differ between performing and observing, and within observing between performing, analytical, and affective orientations. Their coordinated engagement with the conducted performance of ‘Kjempeviseslåtten’ can be understood as an overt enactment of an ‘outward’ connection to the performance. Furthermore, it reveals an embodied, exploratory listening based on being more committed to—*invested in and sensing a responsibility for*—the actions of the musicians on stage. The second listening, in contrast, shows a different trajectory involving expert evaluation of the conductor-less interpretation and the challenges, including rhythmic coordination, facing the musicians. Some listening behaviour exhibited during the second playing of ‘Kjempeviseslåtten’ supports this, with large inspirations directly sympathetic to the oboe soloist (Figure 4d) and exceeding the on-stage conductor’s breathing during the first performance. We can interpret their reflective statements as suggesting a dual intentionality; a close attention to the performance and an inwardly directed connection based on the listener’s own practical understanding of the music, rather than the ‘outward’ focus on preparation and control. This audience member perceived the orchestra as having more freedom in the second performance. Perhaps, the audience member was also liberated by the absence of an on-stage conductor, observing the ensemble without some of the burdens of personal performance association. Such freedom can be seen to be enacted in their continued quiet breathing at rehearsal 6 (Figure 4c), a moment where a conductor would ordinarily take control of the ensemble after solo theme statements.

In summary, we can observe in the spectatorial positions assumed by the audience members an altered responsibility to the music. In other words, we are seeing an awareness on the part of these musician audience members, of a bridging of a gap between the controlled actions of a performer and the affective and reflective explorations afforded in the listening experiences of quiet audience members.

## 5. Conclusions

Our contribution to this special collection presents a data rich approach to researching musical experience ‘in the wild’, integrating physiological measures and phenomenological interviews. Uncovering connections between continuous quantitative measurements and qualitative insights itself depends on collaborative exploration. This extreme closeness of analysis affords a richer understanding of aesthetic experience. Our exploratory analysis of two case studies illuminates idiosyncratic listening trajectories that are shaped by invested attention and action–perception sequences that result from the negotiation of spectatorial and performing roles.

As such, our findings corroborate performing arts research on active, participatory aesthetic engagement, ‘kinesthetic empathy’, and on the spectatorial ‘bridging of the gap’ (Reason & Reynolds, 2010) between audience and performers, but in the particular niche of an orchestral concert. Musically skilled audience members’ bodily relationships with live performances illuminate ways in which practical and affective associations are enacted in the concert hall. Our chosen listening cases also demonstrate forms of exploratory expertise defined by anticipatory capacities and multifaceted forms of intentionality that have elsewhere been reported in music-making settings (Høffding & Schiavio, 2021). The findings presented here also further strengthen the case for ecological–enactive theoretical directions in concert research (Martin & Nielsen, 2024; Høffding et al., 2025) that take seriously the embodied agency of individual participants within aesthetic experience as well as the relational and affective allure of live music encounters.

## Data Availability

Per standard protocol for qualitative research methods, interviews and transcriptions are not publicly available, as they disclose interviewee identity. Quantitative signal features are shared with the analysis and plotting code in the GitHub repository, release v1.0.0 doi:10.5281/zenodo.15458096.

## Notes

1 https://www.uio.no/ritmo/english/projects/Bodies-in-Concert/#:~:text=Concerts%20bring%20audiences%20and%20musicians,of%20qualitative%20and%20quantitative%20methods (accessed on 11 April 2025).

2 We do not know the exact arrangement of ‘Kjempeviseslåtten’ for wind band previously played by this audience member; however, good arrangement practices and this participant’s attention to the low strings suggests a strong correspondence between the Tuba part in wind band and the Contrabass part in this orchestral version, where the actual Tuba part is relatively small. Shading in Figure 1d shows when four relevant parts are active during this piece: the Cello (including the initial solo line) the Contrabass, the Tuba, and the First Trumpet, which playes a fanfare before key iterations of the theme.

## Supplementary Materials

Supporting information can be downloaded at https://www.mdpi.com/article/10.3390/bs15050710/s1.

## Abbreviations

The following abbreviations are used in this manuscript:

SSO	Stavanger Symphony Orchestra
KORK	The Norwegian Radio Orchestra

## References

[B1-behavsci-15-00710] Barker M., Reason M., Lindelof A. M. (2016). Coming a(live): A prolegomenon to any future research on ‘liveness’. Experiencing liveness in contemporary performance: Interdisciplinary perspectives.

[B2-behavsci-15-00710] Bruineberg J., Stone O. (2024). Structuring embodied minds: Attention and perceptual agency. Philosophical Studies.

[B3-behavsci-15-00710] Cannam C., Landone C., Sandler M. (2010). Sonic visualiser: An open source application for viewing, analysing, and annotating music audio files. ACM International Conference on Multimedia.

[B4-behavsci-15-00710] Clarke E. (2005). Ways of listening: An ecological approach to the perception of musical meaning.

[B5-behavsci-15-00710] Clarke E. (2024). What does musicking afford?. Ecological Psychology.

[B6-behavsci-15-00710] Conrad B., Schönle P. (1979). Speech and respiration. Archiv für Psychiatrie und Nervenkrankheiten.

[B7-behavsci-15-00710] Costall A., Ziemke T., Zlatev J., Frank R. M. (2007). Bringing the body back to life: James Gibson’s ecology of agency. Body, language, and mind: Volume 1: Embodiment.

[B8-behavsci-15-00710] Dearn L. K., Pitts S. E. (2017). (Un)popular music and young audiences. Exploring the classical chamber music concert from the perspective of young adult listeners. Journal of Popular Music Education.

[B9-behavsci-15-00710] Di Paolo E., Buhrmann T., Barandiaran X. E. (2017). Sensorimotor life.

[B10-behavsci-15-00710] Dobson M. C., Pitts S. E. (2011). Classical cult or learning community? Exploring new audience members’ social and musical responses to first-time concert attendance. Ethnomusicology Forum.

[B11-behavsci-15-00710] Fadiga L., Tokay S., D’Ausilio A., Flash T., Berthoz A. (2021). Interaction, cooperation and entrainment in music: Experience and perspectives. Space-time geometries for motion and perception in the brain and the arts.

[B12-behavsci-15-00710] Fuchs T. (2017). Ecology of the brain: The phenomenology and biology of the embodied mind.

[B13-behavsci-15-00710] Gallagher S., Bredekamp H., Krois J. (2011). Aesthetics and kinaesthetics. Sehen und handeln.

[B14-behavsci-15-00710] Gallagher S. (2017). Enactivist interventions: Rethinking the mind.

[B15-behavsci-15-00710] Gibson E. J. (2000). Perceptual learning in development: Some basic concepts. Ecological Psychology.

[B16-behavsci-15-00710] Høffding S., Haswell-Martin R., Nielsen N. (2025). Absorbed concert listening: A qualitative, phenomenological inquiry. Philosophies.

[B17-behavsci-15-00710] Høffding S., Heimann K., Martiny K. (2023). Editorial: Working with others’ experience. Phenomenology and the Cognitive Sciences.

[B18-behavsci-15-00710] Høffding S., Martiny K. (2016). Framing a phenomenological interview: What, why and how. Phenomenology and the Cognitive Sciences.

[B19-behavsci-15-00710] Høffding S., Nielsen N., Laeng B. (2024). Mind surfing: Attention in musical absorption. Cognitive Systems Research.

[B20-behavsci-15-00710] Høffding S., Schiavio A. (2021). Exploratory expertise and the dual intentionality of music-making. Phenomenology and the Cognitive Sciences.

[B21-behavsci-15-00710] Kozak M. (2019). Enacting musical time: The bodily experience of new music.

[B22-behavsci-15-00710] Køster A., Fernandez A. V. (2023). Investigating modes of being in the world: An introduction to Phenomenologically grounded qualitative research. Phenomenology and the Cognitive Sciences.

[B23-behavsci-15-00710] Martin R., Nielsen N. (2024). Enacting musical aesthetics: The embodied experience of live music. Music & Science.

[B24-behavsci-15-00710] Matyja J. R., Schiavio A. (2013). Enactive music cognition: Background and research themes. Constructivist Foundations.

[B25-behavsci-15-00710] McFee B., McVicar M., Faronbi D., Roman I., Gover, M., Balke S., Seyfarth S., Malek A., Raffel C., Lostanlen V., van Niekirk B., Lee D., Cwitkowitz F., Zalkow F., Nieto O., Ellis D., Mason J., Lee K., Steers B., Pimenta W. (2023). librosa/librosa: 0.10.1 *(0.10.1)*.

[B26-behavsci-15-00710] Merrill J., Czepiel A., Fink L. T., Toelle J., Wald-Fuhrmann M. (2023). The aesthetic experience of live concerts: Self-reports and psychophysiology. Psychology of Aesthetics, Creativity, and the Arts.

[B27-behavsci-15-00710] Noë A. (2006). Action in perception.

[B28-behavsci-15-00710] O’Neill K., Egermann H. (2022). Development of the social experience of a concert scales (SECS): The social experience of a live western art music concert influences people’s overall enjoyment of an event but not their emotional response to the music. Music & Science.

[B29-behavsci-15-00710] Pitts S. E. (2005). What makes an audience? Investigating the roles and experiences of listeners at a chamber music festival. Music and Letters.

[B30-behavsci-15-00710] Pitts S. E., Price S. M. (2021). Are you being engaged? Exploring the limits of audience capacity for engagement. Journal of Audience and Reception Studies.

[B31-behavsci-15-00710] Prätzlich T., Driedger J., Müller M. (2016). Memory-restricted multiscale dynamic time warping. 2016 IEEE International Conference on Acoustics, Speech and Signal Processing (ICASSP).

[B32-behavsci-15-00710] Read C., Szokolszky A. (2020). Ecological psychology and enactivism: Perceptually-guided action vs. sensation-based enaction. Frontiers in Psychology.

[B33-behavsci-15-00710] Reason M., Conner L., Johanson K., Walmsley B., Reason M., Conner L., Johanson K., Walmsley B. (2022). The paradox of audiences. Routledge companion to audiences and the performing arts.

[B34-behavsci-15-00710] Reason M., Reynolds D. (2010). Kinesthesia, empathy, and related Pleasures: An inquiry into audience experiences of watching dance. Dance Research Journal.

[B35-behavsci-15-00710] Reybrouck M., Podlipniak P., Welch D. (2024). Music listening as exploratory behavior: From dispositional reactions to epistemic interactions with the sonic world. Behavioral Sciences.

[B36-behavsci-15-00710] Small C. (1998). Musicking: The meanings of performing and listening.

[B37-behavsci-15-00710] Swarbrick D., Martin R., Høffding S., Nielsen N., Vuoskoski J. K. (2024). Audience musical absorption: Exploring attention and affect in the live concert setting. Music & Science.

[B38-behavsci-15-00710] Swarbrick D., Vuoskoski J. K. (2023). Collectively classical: Connectedness, awe, feeling moved, and motion at a live and livestreamed concert. Music & Science.

[B39-behavsci-15-00710] Tröndle M., Bishop E., Tröndle M. (2021). Concert studies. Classical concert studies: A companion to contemporary research and performance.

[B40-behavsci-15-00710] Upham F. (2018). Detecting the adaptation of listeners’ respiration to heard music. Ph.D. Dissertation.

[B41-behavsci-15-00710] Upham F. (2025). Exploratory Listening Physiology Analysis *(Version 1.0.0) [Computer software]*.

[B42-behavsci-15-00710] Vlemincx E., Abelson J. L., Lehrer P. M., Davenport P. W., Van Diest I., Van den Bergh O. (2013). Respiratory variability and sighing: A psychophysiological reset model. Biological Psychology.

[B43-behavsci-15-00710] Wald-Fuhrmann M., Egermann H., Czepiel A., O’Neill K., Weining C., Meier D., Tschacher W., Uhde F., Toelle J., Tröndle M. (2021). Music listening in classical concerts: Theory, literature review, and research program. Frontiers in Psychology.

[B44-behavsci-15-00710] Zagorski-Thomas S. (2014). The musicology of record production.

[B45-behavsci-15-00710] Zatorre R. J., Chen J. L., Penhune V. B. (2007). When the brain plays music: Auditory-motor interactions in music perception and production. Nature Reviews Neuroscience.

