# Supplementary material for: Embodied, Exploratory Listening in the Concert Hall
Source: Behav Sci (Basel). 2025 May 21;15(5):710. doi: 10.3390/bs15050710 (PMC12109233; doi:10.3390/bs15050710)
Supplement: Supplementary file 1 [file behavsci-15-00710-s001.zip › behavsci-3528724-supplementary.pdf]

Supplementary materials

Case Study 1, other audience measurements

Respiration sequences from 18 music student participants during SSO performances of Kjempe. The proportion of quiet breathing time was in the 60-80% range, with the featured audience member at the high end, by this definition of quiet breathing.

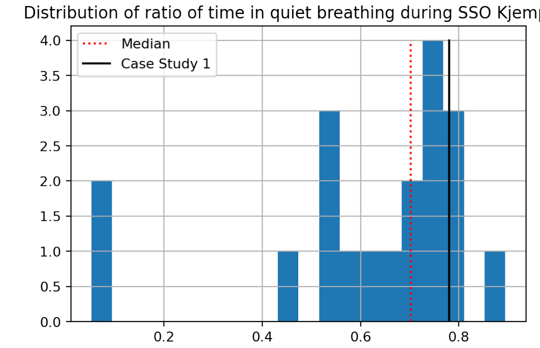

To assess the likelihood of the coincidences of audience member expiration onsets with identified music moments, phase onsets from each sequence were extracted and time from onsets time series generated, as below.

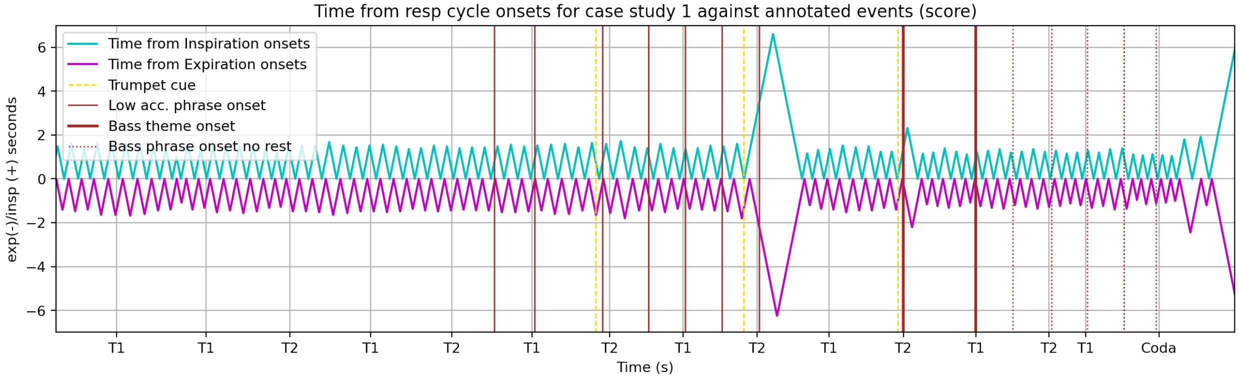

The cumulative distribution of distances from audience member expiration onsets to the 8 moments identified as bass line entries suitable for inspirations. On the left, are the distributions when shifted from the original temporal alignment (+/-12 s, sampled at 0.3 s). On the right are the distances to onset from other audience members all in original timing.

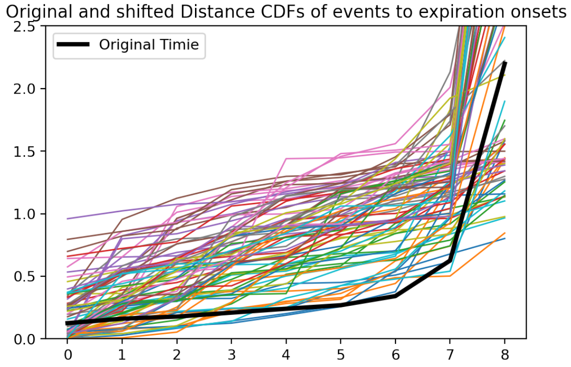

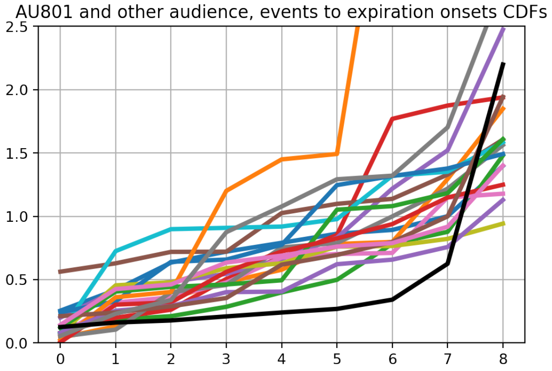

Case Study 2, Other audience member

Respiration sequences from 15 adult audience members, including 5 music student participants during the two performances of Kjempe…. Unlike in case study 1, this audience member shows less quiet breathing than most audience participants, 47% of time to a median of 63% of time through the first performance of this work and 63% to a median 74 % of time through the second.

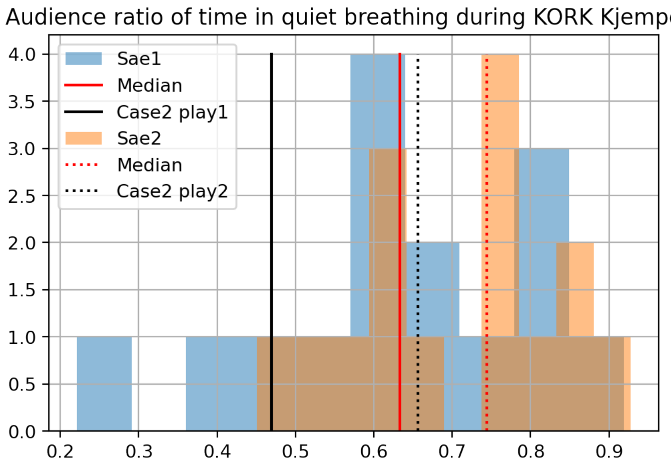

The cumulative distributions of the quantity of motion (jerk magnitude) from each participant during the two performances and the body motion from the on stage conductor during the first performances

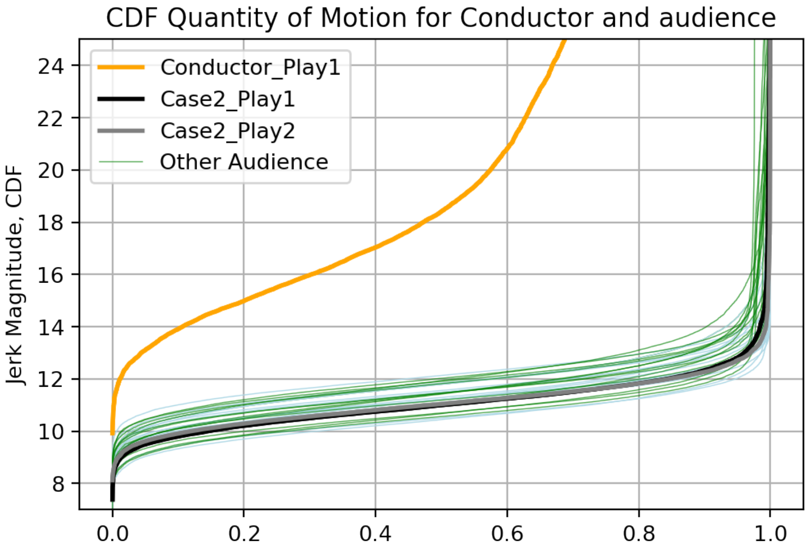

Computation and generation of these figures retrievable from https://github.com/finn42/Exploratory_Listening_Phys
